# Michel Wensing, Charlotte Ullrich, editors: Foundation of Health Services Research. Principles, Methods, and Topics

**DOI:** 10.3205/zma001690

**Published:** 2024-09-16

**Authors:** Elisabeth Schmidt

**Affiliations:** 1 Martin-Luther-University Halle-Wittenberg, Medical Faculty Halle-Wittenberg, Dorothea Erxleben Learning Center Halle, Halle/Saale, Germany

## Bibliographical details

Michel Wensing, Charlotte Ullrich, editors

**Foundation of Health Services Research. Principles, Methods, and Topics**

Publisher: Springer Nature Switzerland

Year of publication: 2023, 317 pages, price: Hardcover: € 117.89, eBook: € 93.08

ISBNs: 978-3-031-29997-1, 978-3-031-29998-8

## Review

Medical and health research often takes place under laboratory conditions and not in everyday practice. However, many topics for improving and optimizing healthcare arise from everyday clinical practice. They need to be researched and answered under real-life conditions so that they can be implemented in everyday healthcare. Health services research deals with research questions on health care under real-world conditions and looks at both the results and the determinants of health care. Three levels are considered here: the micro level of interaction between patient and healthcare provider, the meso level of healthcare facilities and the macro level of the healthcare system at regional, national or international level. Possible questions in healthcare research could be, for example, how unnecessary prescribing of opioids can be reduced or how the transition from inpatient to outpatient care can be coordinated.

The book “Foundation of Health Services Research. Principles, Methods, and Topics” describes basics of health services research including principles, methods and (future) topics. The book is divided into four parts and 24 chapters.

The chapters are well organized and presented in a logical order. Practical examples are given throughout. All chapters are preceded by an abstract, which provides an overview of the content. Relevant studies on the topic are briefly presented and core statements of the respective chapter are highlighted each in gray boxes. Many tables are used to relate key content. The clear structure of the book and the many tables and overviews make it possible to quickly grasp the relevant content and, above all, its interrelationships. The book is also very clear thanks to the practical examples. In addition to the authors' references to the literature, there are references to additional in-depth literature. The connection to healthcare research and healthcare practice is very well developed and presented in all chapters. The book reads well and is easy to follow. 

The first part *(chapters 1-2)* describes health services research, including its definition, objectives and fields. Health services research is also differentiated from basic and clinical research. The second part *(chapters 3-6)* focuses on the principles of health services research, including theories, scientific integrity and the presentation of quantitative research results. The transfer of research results into health care and the dissemination of research results are also discussed, which in health services research goes far beyond scientific publications in order to reach the relevant target groups and stakeholders. The various methods used in health services research are presented in the third part *(chapters 7-16)* of the book. Health services research uses existing research methods, which are adapted to the context. With regard to health services research, qualitative research methods, survey methods, but also network analysis, questionnaire development and validation as well as outcome and process evaluation in health services research are discussed and illustrated with corresponding examples. The fourth part *(chapters 17-24)* of the book presents forward-looking topics in healthcare research, such as new interventions for patient empowerment, digital technologies for information and communication in healthcare or climate change as a future topic for health services research.

The motivation of the editors to publish this book results from the university teaching of a master’s degree course in health services research. The aim of the book is to provide an introduction and an overview of health services research. The book is primarily aimed at students and newcomers to health services research. The book fully meets this requirement thanks to the appropriate selection of topics, practical references and the stringent logic of the book. Only the high price is not very student-friendly. Even those already familiar with the subject of health services research will find new insights and connections. The book is also helpful for qualification work in the field. The book can also serve as a decision-making aid for questions or methodological approaches. Anyone wishing to use a corresponding method will have to delve deeper into further (methodological) literature. The fourth part of the book in particular can serve as a source of inspiration for one’s own research ideas and thus contribute to the long-term improvement of health care processes.

## Competing interests

The author declares that she has no competing interests.

